# Economic evaluation of sea-level rise adaptation strongly influenced by hydrodynamic feedbacks

**DOI:** 10.1073/pnas.2025961118

**Published:** 2021-07-12

**Authors:** Michelle A. Hummel, Robert Griffin, Katie Arkema, Anne D. Guerry

**Affiliations:** ^a^Department of Civil Engineering, University of Texas at Arlington, Arlington, TX 76019;; ^b^The Natural Capital Project, Stanford University, Stanford, CA 94305;; ^c^School for Marine Science and Technology, University of Massachusetts Dartmouth, Dartmouth, MA 02747;; ^d^School of Environmental and Forest Sciences, University of Washington, Seattle, WA 98195

**Keywords:** sea-level rise, adaptation, economic damages, externalities, flooding

## Abstract

Coastal communities rely on levees and seawalls as critical protection against sea-level rise; in the United States alone, $300 billion in shoreline armoring costs are forecast by 2100. However, despite the local flood risk reduction benefits, these structures can exacerbate flooding and associated damages along other parts of the shoreline—particularly in coastal bays and estuaries, where nearly 500 million people globally are at risk from sea-level rise. The magnitude and spatial distribution of the economic impact of this dynamic, however, are poorly understood. Here we combine hydrodynamic and economic models to assess the extent of both local and regional flooding and damages expected from a range of shoreline protection and sea-level rise scenarios in San Francisco Bay, California. We find that protection of individual shoreline segments (5 to 75 km) can increase flooding in other areas by as much as 36 million m^3^ and damages by $723 million for a single flood event and in some cases can even cause regional flood damages that exceed the local damages prevented from protection. We also demonstrate that strategic flooding of certain shoreline segments, such as those with gradually sloping baylands and space for water storage, can help alleviate flooding and damages along other stretches of the coastline. By matching the scale of the economic assessment to the scale of the threat, we reveal the previously uncounted costs associated with uncoordinated adaptation actions and demonstrate that a regional planning perspective is essential for reducing shared risk and wisely spending adaptation resources in coastal bays.

## Significance

As sea levels rise, coastal cities will rely on shoreline protection strategies such as levees and seawalls to mitigate flooding. Although these strategies provide local flood-reduction benefits, they can increase inundation along other shorelines within the same estuary or bay. Using hydrodynamic and economic models, we quantify previously unmeasured regional economic damages (up to $723 million per flood event) that result from the protection of individual shoreline segments in San Francisco Bay, CA. We also highlight and quantify opportunities to alleviate regional flood damage through strategic floodwater storage in low-lying areas. Integrating the findings into coordinated planning efforts that account for the regional impacts of local shoreline actions could provide opportunities to reduce shared risk in coastal regions globally.

Sea-level rise (SLR) threatens to produce more frequent and severe flooding in coastal regions and is expected to cause trillions of dollars in damages globally by 2100 if society does not take action to adapt to this threat (1). Lives and livelihoods are at risk as well; globally, hundreds of millions of people could be exposed to SLR by 2100 (2–4). A critical challenge in responding to this threat is that decisions about strategies for adaptation to coastal flooding are often made by individual communities or private entities with limited cross-jurisdictional coordination and at a scale that does not match the hydrodynamic extent of the threat (5–7). Populated coastal areas are coupled human–natural systems, where spatial and temporal interactions between hydrodynamics and shoreline modification influence patterns of flooding, erosion, and resulting damage to communities (8, 9). In these settings, individual action tends to impact other parties (externalities) and yield outcomes different from those that would arise from collective decision-making (10), generally resulting in reduced overall social welfare (11). Even so, collective approaches to shoreline adaptation are often hindered by existing governance structures that rely on local oversight of coastal management or fragmented approaches to project permitting and implementation (7).

Spatial externalities are common in coupled human–natural systems. High-profile examples include the “dead zone” in the Gulf of Mexico and its link to upstream nutrient runoff from agriculture carried down the Mississippi, widespread acid rain in the northeastern United States originating from power plants in the Midwest that led to revisions of the Clean Air Act in 1990, and the visual impacts on adjacent property owners from the Cape Wind offshore wind farm near Nantucket, MA, that led to its eventual demise after more than a decade of litigation. Spatial externalities are also common and varied in the context of shoreline protection and management. In river systems, it has long been known that channel modifications and levee building at one location can influence water levels and flood potential at locations both upstream and downstream (12–15). On open coasts, alongshore currents can affect the efficacy of beach nourishment projects through mobilization and loss of sediment to neighboring beaches (16, 17). As a result, individual communities may be incentivized to nourish their beaches less frequently, either to avoid paying for sediment that is subsequently lost to undernourished beaches in neighboring communities or in the hopes of benefiting from sediment input from nourishment projects elsewhere (18). Waves can also interact with protection structures to induce erosion in adjacent areas (19). A recent study found that these interactions reduced property values for adjacent shoreline properties that are ineligible to build their own protection structures by 8% on average in coastal Oregon (20).

Shoreline armoring will play a key role in responding to SLR moving forward. It is forecast to represent nearly 60% of the roughly $500 billion in US adaptation costs by 2100 (21). Despite evidence for a wide range of spillover effects resulting from shoreline modification and the billions in planned expenditures on these modifications, there is limited understanding about how they influence shared economic risk across the coastal zone (5). Erosion and beach nourishment are better understood than coastal flooding, where the only economic assessment of externalities is on the performance of critical infrastructure systems (22, 23).

To address these gaps and account for the physical and economic impacts of flooding on communities, here we couple dynamic simulations of coastal inundation with models of building damage to examine flood damage externalities expected under a range of shoreline modification and SLR scenarios. We focus on the densely populated San Francisco Bay Area, as bay and estuarine systems in particular are characteristic of coastal locations that feature regional coastal hydrodynamic interactions. In these settings, engineered protection can lead to amplification of water levels, cause additional flooding in other locations, and in some cases adversely affect coastal vegetation and the shoreline protection benefits it provides (24, 25). Conversely, shoreline modification to strategically store water can have the opposite effect, providing dissipation that attenuates water levels and produces regional flood reduction benefits (26–30). Bays and estuaries represent 21% of overall shoreline length and 54% of global population at risk from SLR and flooding—nearly half a billion people (see *Materials and Methods*). These densely populated areas with complex jurisdictional boundaries are increasingly facing difficult and expensive decisions that demand a better understanding of shared risk along the coastline.

## Approach

**Fig. 1.**
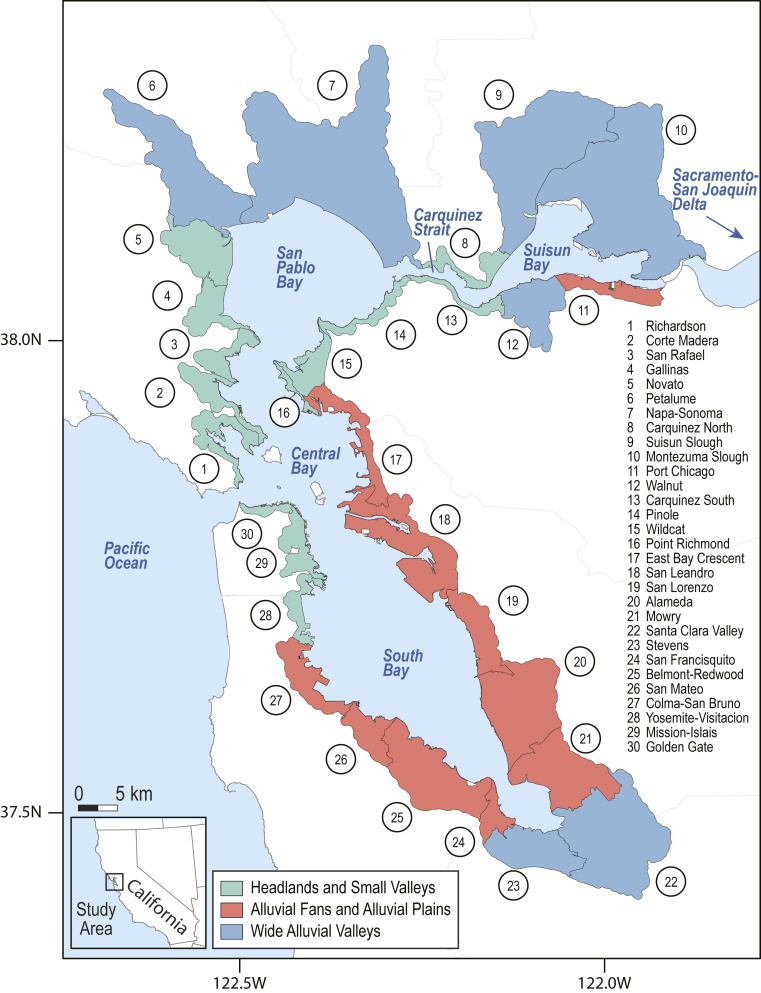
Map of the San Francisco Bay Area, showing the 30 OLUs developed by ref. 35, their geomorphic classifications, and their names.

San Francisco Bay is the largest coastal embayment in California and is composed of four distinct subembayments: Suisun Bay, San Pablo Bay, Central Bay, and South Bay (Fig. 1). Buildings adjacent to the bay that are exposed to the effects of SLR over the next 150 y represent more than $180 billion in replacement value and are home to a population of over 1.4 million people (see *Materials and Methods*). Together, the nine counties that surround San Francisco Bay represent the majority of population and building exposure to coastal flooding in California (31). Shoreline modification is widespread throughout the bay, with 6% of the shoreline behind levees designed specifically for flood protection and 75% of the shoreline modified as berms, embankments, transportation infrastructure, or other engineering that affects flooding and flood routing (32). Recent modeling studies of shoreline adaptation and SLR in San Francisco Bay have demonstrated that shoreline protection using engineered structures like seawalls can cause amplification of the tides by reducing frictional damping in shallow areas along the perimeter of the bay and enhancing reflection of the incoming tidal wave at the shoreline (26, 28, 29). These changes in tidal amplitude can influence the magnitude and spatial distribution of peak water levels and inundation around the bay.

To assess the distribution of regional economic impacts associated with local-scale shoreline protection from SLR, we quantify the spatial change in inundation and economic damages from implementing protection strategies in the San Francisco Bay Area under four SLR scenarios (50, 100, 150, and 200 cm above a January 2010 baseline). SLR projections for San Francisco Bay suggest a likely range (67% probability) of 30 to 104 cm of SLR above the 1991 to 2009 mean by 2100 (33), although SLR exceeding 200 cm is also possible under rapid Antarctic ice-sheet melt (34). For each SLR scenario we simulate an existing shoreline scenario that includes all present-day infrastructure, as well as 30 shoreline modification scenarios in which a single segment of the shoreline is completely protected by a seawall while the rest of the shoreline is maintained as is, such that it remains vulnerable to flooding where not currently protected. For all SLR and shoreline modification scenarios we assume no landward migration of the shoreline. The 30 shoreline segments are based on operational landscape units (OLUs) delineated by ref. 35 along the San Francisco Bay shoreline to inform SLR adaptation planning (Fig. 1). These OLUs represent terrestrial and coastal regions, ranging in coastline length from 5 to 75 km, with similar physical and ecological processes that together provide a cohesive set of ecosystem functions and similar adaptation possibilities (36). These are classified into one of three geomorphic categories that account for the geologic history of the region and its influence on landscape features. Wide alluvial valleys are characterized by wide baylands and gradual slopes, alluvial fans and alluvial plains consist of baylands of intermediate width and moderate slopes, and headlands and small valleys exhibit narrow baylands and steep slopes (35) (*SI Appendix*, Fig. S1).

We use a two-dimensional, depth-averaged hydrodynamic model of San Francisco Bay (37, 38) to simulate tidal circulation and interactions with the bay shorelines for each scenario (see *Materials and Methods*). Changes in tidal dynamics and bay water levels resulting from these modeled scenarios are described in ref. 29. Here, we extract spatially varying maximum water depths from the model at high tide during a spring tide cycle to capture inundation in areas that experience tidal flooding or permanent inundation. We integrate these values across the land area to find the total volume of flood water in each OLU in each scenario. Comparing this flood volume to similarly derived volume estimates for the existing shoreline scenario at the same SLR provides spatially explicit estimates of internal (within the protected OLU) and external (in other OLUs) tidal flooding for each shoreline protection scenario. To estimate associated economic impacts from this flooding, we overlay the flood depth maps with building stock data from the HAZUS flood model (39) and use depth-damage curves to compute changes in damages between the existing and protected shoreline scenarios for a one-off flood event. We use the existing building stock data to estimate economic damages and do not attempt to forecast future changes in land use or shoreline habitat distribution in the region. The combined flood and damage results allow for an analysis of the spatial extent of interactions, from local effects on neighboring OLUs in the same subembayment to regional or baywide effects.

## Effect of Shoreline Protection Scenarios on Inundation

### OLU Interactions.

**Fig. 2.**
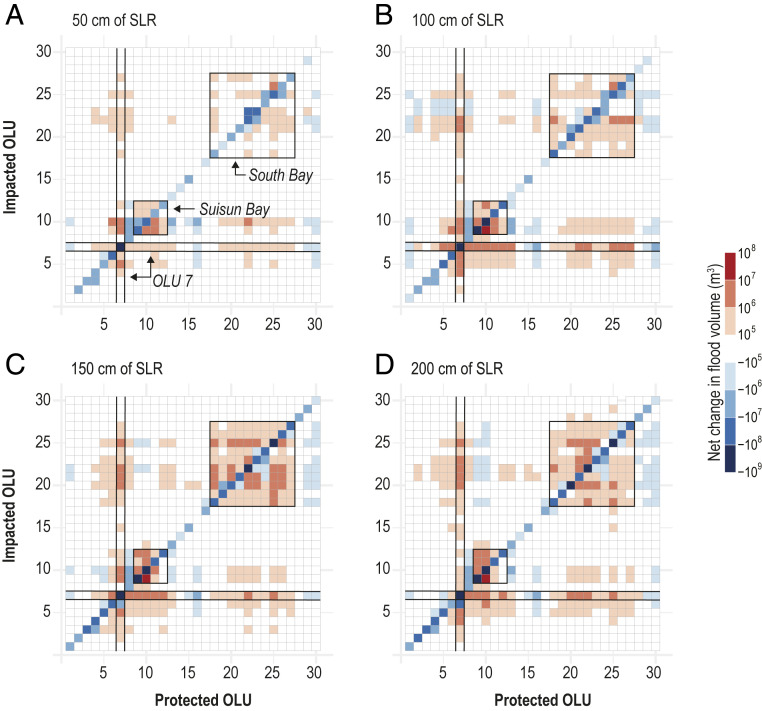
Net change in flood volume for OLU protection scenarios at (*A*) 50 cm, (*B*) 100 cm, (*C*) 150 cm, and (*D*) 200 cm of SLR. Individual OLU protection scenarios are along the horizontal axis. Each column shows the net change in flood volume in all other OLUs resulting from that protection scenario. OLU numbering is shown in Fig. 1. Subembayment interactions in Suisun Bay and South Bay are indicated by boxes. OLU 7 (Napa–Sonoma), which experiences strong interactions with other OLUs under shoreline protection scenarios, is also highlighted.

Fig. 2 summarizes the flood impacts due to the modeled shoreline protection scenarios at (*A*) 50 cm, (*B*) 100 cm, (*C*) 150 cm, and (*D*) 200 cm of SLR. The OLU protection scenarios are listed along the horizontal axis. Each column shows the net change in flood volume in all other OLUs resulting from that protection scenario. OLU numbering is shown in Fig. 1. Values along the diagonal represent the reduction in internal flooding in the protected OLU as compared to the existing shoreline scenario and range from −1,900 m^3^ for OLU 30 (Golden Gate) at 50 cm of SLR to −551 million m^3^ for OLU 7 (Napa–Sonoma) at 200 cm of SLR.

Off-diagonal values represent protection-induced external flooding in other OLUs, which is generally greatest between OLUs in the same subembayment. In Suisun Bay (OLUs 9 to 12), protection and subsequent loss of floodwater storage capacity in any one OLU typically leads to an increase in flooding in other OLUs. For example, when OLU 10 (Montezuma Slough) protects its shoreline, flooding in OLU 9 (Suisun Slough) increases by almost 30 million m^3^ at 100 cm of SLR, as water that formerly flooded OLU 10 is redirected elsewhere. In South Bay (OLUs 18 to 27), protection of certain OLUs similarly exacerbates flooding in other South Bay OLUs, although the magnitude of interactions is smaller, with a maximum increase of 4.2 million m^3^ of flooding in OLU 20 (Alameda) due to protection of OLU 22 (Santa Clara Valley) at 200 cm of SLR.

**Fig. 3.**
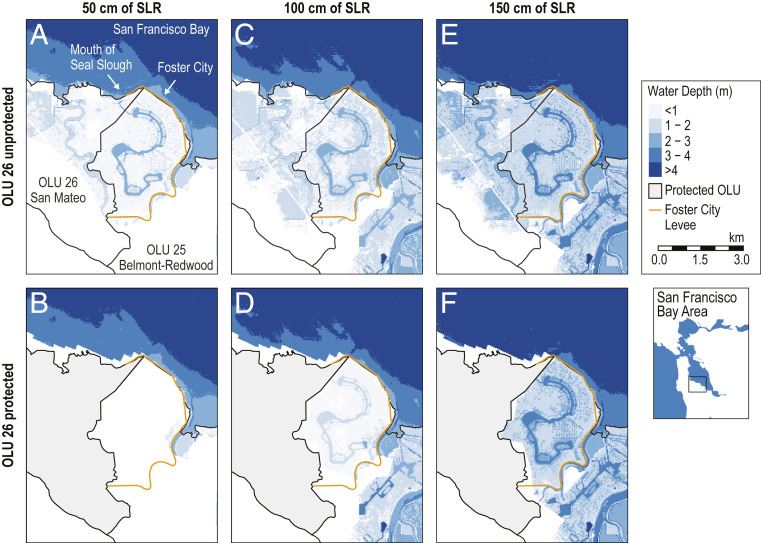
Interaction between OLUs 25 (Belmont–Redwood) and 26 (San Mateo) in South San Francisco Bay. At 50 cm of SLR (*A* and *B*), protection of the OLU 26 shoreline eliminates the flood pathway at the mouth of Seal Slough, such that Foster City, located behind the levee in OLU 25, remains dry. At 100 cm of SLR (*C* and *D*), the Foster City levee is overtopped, so protection of the OLU 26 shoreline provides only partial flood reduction in OLU 25. At 150 cm of SLR (*E* and *F*), protecting the OLU 26 shoreline causes additional flooding in OLU 25. The 200-cm SLR scenario shows the same interaction as the 150-cm scenario and thus is not included here.

Notably, protection of South Bay OLUs can lead to a reduction in flooding in neighboring OLUs under certain SLR scenarios (Fig. 2), as flood pathways across lateral OLU boundaries stretching inland from the coast are eliminated. For example, Foster City, which is part of OLU 25 (Belmont–Redwood) (Fig. 3), is surrounded by a levee that provides full protection from direct coastal flooding at 50 cm of SLR. However, the elevated sea level pushes additional water into the mouth of a neighboring channel, Seal Slough, along the shoreline of OLU 26 (San Mateo), which leads to widespread flooding behind the levee in OLU 25 (Fig. 3*A*). With protection of the shoreline of OLU 26 comes elimination of the flood pathway at the mouth of Seal Slough, such that Foster City remains dry (Fig. 3*B*), leading to a reduction of 6.5 million m^3^ of flooding for OLU 25 due to protection in OLU 26. At 100 cm of SLR, parts of the Foster City levee are overtopped, causing direct flooding along the shoreline of OLU 25 (Fig. 3*C*). However, protecting OLU 26 still provides substantial benefits for OLU 25 (Fig. 3*D*), reducing flooding by 5.5 million m^3^. At 150 cm of SLR and higher these benefits are lost; protecting OLU 26 leads to an additional 1.1 million m^3^ of flooding in OLU 25 (Fig. 3*F*) compared with the existing shoreline scenario (Fig. 3*E*). As this example demonstrates, the external impact of shoreline protection may change over time as SLR progresses.

Some OLU protection scenarios also cause external flooding that extends regionally to other subembayments. These cross-embayment interactions are most notable between OLU 7 in San Pablo Bay and OLUs in Suisun Bay and South Bay (Fig. 2). In both cases, the physical characteristics and geographic location of OLU 7 play an important role in its relationship to regional inundation patterns. When OLUs in Suisun Bay are protected, tides propagating from the ocean inlet landward toward the Sacramento–San Joaquin Delta interact with the shoreline infrastructure to create feedbacks that affect the down-estuary (seaward) water level response and cause additional flooding in OLU 7. Protecting the OLU 7 shoreline similarly leads to additional flooding up-estuary (landward) in Suisun Bay, particularly in OLUs 9 and 10. The relationship between South Bay OLUs and OLU 7 is also bidirectional. For example, protection of South Bay shorelines (OLUs 20 to 22 and 25 to 27) causes additional inundation in OLU 7. Similarly, when OLU 7 is protected, flooding is exacerbated in several South Bay OLUs, most notably OLUs 20 to 23 and 25. OLU 7’s low elevation and large area provide substantial storage space for floodwaters when shorelines are not modified, but this space is lost when protection is implemented along its shoreline. Unlike OLUs 9 and 10, which provide similar storage space but are separated from the rest of the bay via the narrow Carquinez Strait, OLU 7’s position at the northern boundary of the bay leads to changes in down-estuary water levels in San Pablo Bay and Central Bay that propagate into South Bay.

### Geomorphic Influence.

**Fig. 4.**
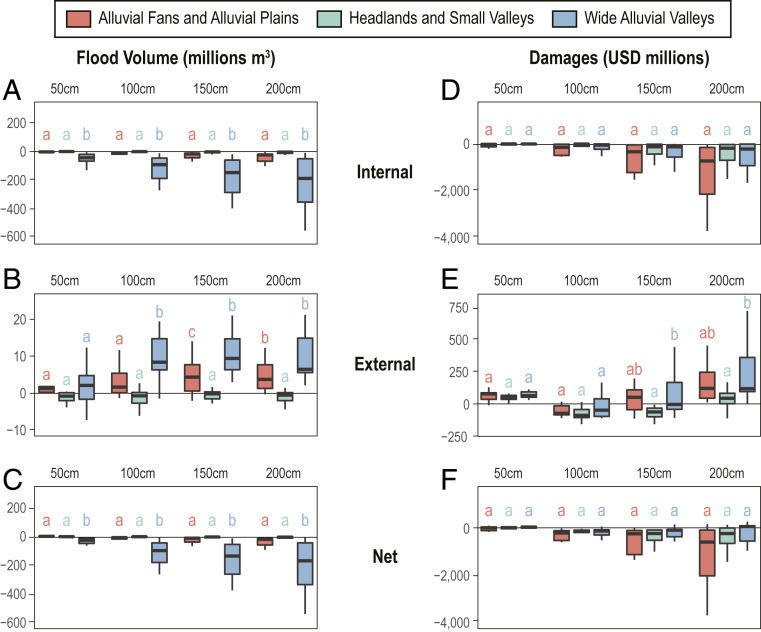
Distribution of OLU flood and damage results, summarized by geomorphic type. (*Left*) The change in (*A*) internal flooding within the protected OLU, (*B*) external flooding across all other OLUs, and (*C*) net flooding (internal plus external) with protection. (*Right*) The change in (*D*) internal damages within the protected OLU, (*E*) external damages across all other OLUs, and (*F*) net damages (internal plus external) with protection. Letters represent statistically similar mean values for all pairwise comparisons across geomorphic types within a SLR scenario, based on a Tukey honest significant difference test at 5% significance level. The significance test results for all pairwise comparisons are provided in *SI Appendix*, Table S1.

Geomorphic characteristics play an important role in determining the internal and external impacts of shoreline protection. Large decreases in internal flooding result from protection of OLUs classified as wide alluvial valleys, as low-lying areas are disconnected from the bay (Fig. 4*A*). Alluvial fans and alluvial plains and headlands and small valleys experience smaller decreases. Increases in external flooding are also generally largest for protection of wide alluvial valleys and least for headlands and small valleys. The low elevations and gradual slopes that characterize wide alluvial valleys can provide frictional damping of the tides (29) and store floodwaters more readily than other geomorphic types. However, when the shoreline is protected, this storage space is lost and the OLU boundary shifts from dissipative to reflective, leading to tidal amplification within the bay (29) and exacerbating flooding in other OLUs. In contrast, protection of certain headlands and small valleys leads to small decreases in external flooding, indicating the potential for a regional benefit to protecting these areas (Fig. 4*C*). Because these OLUs are typically located at narrower parts of the bay, shoreline protection leads to additional narrowing that may slightly reduce tidal energy transmission through these areas. For example, protecting OLUs 8 (Carquinez North) and 13 (Carquinez South) along the Carquinez Strait leads to a reduction in up-estuary flooding in OLUs 9 to 12 surrounding Suisun Bay (Fig. 2), as less water is able to move through the constricted channel into Suisun Bay during the tidal cycle. Overall, reductions in internal flooding due to shoreline protection are generally greater than increases in induced external flooding, resulting in a net decrease, regionally, in flood volume for almost all OLU shoreline protection scenarios across all three geomorphic types (Fig. 4*E*).

## Economic Damages Due to Coastal Inundation

### OLU Interactions.

**Fig. 5.**
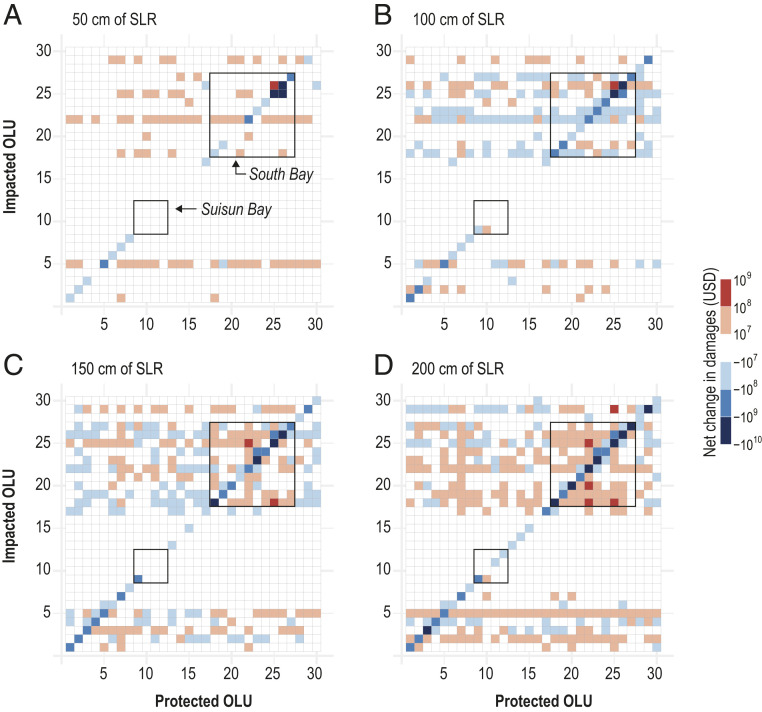
Net change in economic damages for OLU protection scenarios at (*A*) 50 cm, (*B*) 100 cm, (*C*) 150 cm, and (*D*) 200 cm of SLR. The OLU protection scenarios are listed along the horizontal axis. Each column shows the net change in economic damages in all other OLUs resulting from that protection scenario. OLU numbering is shown in Fig. 1. Subembayment interactions in South Bay and Suisun Bay are indicated by the boxes in each plot.

Fig. 5 summarizes the damage interactions resulting from the modeled shoreline protection scenarios at (*A*) 50 cm, (*B*) 100 cm, (*C*) 150 cm, and (*D*) 200 cm of SLR. Internal reductions in economic damages, shown along the diagonal, are generally largest in the South Bay (OLUs 18 to 27), where dense development lies right along the shoreline. In OLU 25 alone, internal damages are reduced by $1.4 to 6.1 billion across the four SLR scenarios when the shoreline is protected. Internal benefits are smallest along the southern extent of Suisun Bay, the Carquinez Strait, and San Pablo Bay (OLUs 11 to 16), ranging from $0.4 to 55 million.

In contrast to the flooding results, which exhibit strong external interactions between OLUs in Suisun Bay, the damage interactions within Suisun Bay are not as notable. Development in this region is more sparse compared to parts of Central and South Bay, and large portions of the shoreline consist of wetlands, leading to relatively low building replacement costs per unit area (*SI Appendix*, Table S2) and limiting the potential magnitude of damage externalities. On the other hand, protecting South Bay OLUs leads to large external damages in other OLUs in South Bay (top right of Fig. 5 *A*–*D*), which become more pronounced and widespread at higher sea levels. These externalities primarily result in increased damages in other OLUs at 50 cm, 150 cm, and 200 cm of SLR (Fig. 5 *A*, *C*, and *D*), except for adjacent OLUs, which may experience damage reductions due to lateral flood protection. Damage externalities are especially notable for the OLU 22 protection scenario, which leads to additional damages in all other South Bay OLUs, totaling $723 million at 200 cm of SLR. In contrast, South Bay interactions at 100 cm of SLR lead to generally small but widespread damage reductions that are not limited to adjacent OLUs (Fig. 5*B*). For example, protecting OLU 20 provides flood reduction benefits for its neighbors, OLUs 19 (San Lorenzo) and 21 (Mowry), but also for OLUs 23 (Stevens) and 24 (San Francisquito) on the opposite shoreline. Thus, while the flooding results show a more consistent pattern of increasing external flood volume across all SLR scenarios (Fig. 2), the damage results exhibit greater variation, as they are a function of both the hydrodynamic–shoreline interactions that govern flooding as well as the spatial distribution of development and high-value properties.

Regional external damage interactions are also present in some protection scenarios, especially at higher sea levels (Fig. 5 *C* and *D*). When OLU 7 is protected at 200 cm of SLR, OLUs 22 and 18 (San Leandro) in South Bay experience an additional $82 million and $70 million in damages, respectively, while OLU 3 (San Rafael) in San Pablo Bay experiences an additional $53 million in damages (Fig. 5*D*). OLU 22, with the highest building replacement cost for a wide alluvial valley in the bay (*SI Appendix*, Table S2), is susceptible to damage interactions with nearly every external protection scenario at 200 cm of SLR.

While the focus of our analysis is on damage to structures, population impacts are another important consideration when developing shoreline adaptation strategies. The individual shoreline protection scenarios considered here can cause as many as 5,900 additional people to be affected by external flooding (*SI Appendix*, Table S3), as is the case when OLU 22 is protected. We provide an example of how population impacts could be used to supplement economic damage data in *Discussion*.

### Geomorphic Influence.

Differences between OLU geomorphic classifications are more muted for economic damages than for flood volume. Estimated reductions in internal economic damages appear greatest in OLUs classified as alluvial fans and alluvial plains (Fig. 4*D*), though this is not statistically significant for any pairwise comparison. Surprisingly, the large internal flood reductions estimated for protecting wide alluvial valleys do not translate to similarly large damage reductions. The coastal landscape configuration in this type of OLU is generally a mix of coastal wetlands, grassland, and pasture land (based on 2016 National Land Cover Database; *SI Appendix*, Fig. S2) that limits exposure of development to flooding. While external damage patterns for protecting wide alluvial valleys are qualitatively consistent with external flood patterns by geomorphic type, most of these relationships are not statistically significant, with the exception of observed greater external damages than headlands and small valleys under 150 cm and 200 cm of SLR (Fig. 4*E*).

## Discussion

Regional externalities resulting from hydrodynamic feedbacks are an important consideration when evaluating protection strategies in highly developed coastal embayments. Although there are large potential benefits from avoided flood damage behind protective infrastructure in the San Francisco Bay Area, this analysis shows that these benefits can come at a cost to other shoreline communities, both nearby and in other parts of the bay. The increase in baywide inundation volume and external damages that results from the protection of a single OLU can be as large as 36 million m^3^ and $723 million, respectively. Assessing flood patterns by geomorphic type, we identify factors that contribute to external changes in flood volume from protection, including space for water storage and proximity to narrow straits. While these factors extend to other coastal embayments, external changes in flood damage rely on the spatial distribution and overlap of flooding and exposed buildings and will require a model like we have introduced here to estimate these impacts elsewhere.

**Fig. 6.**
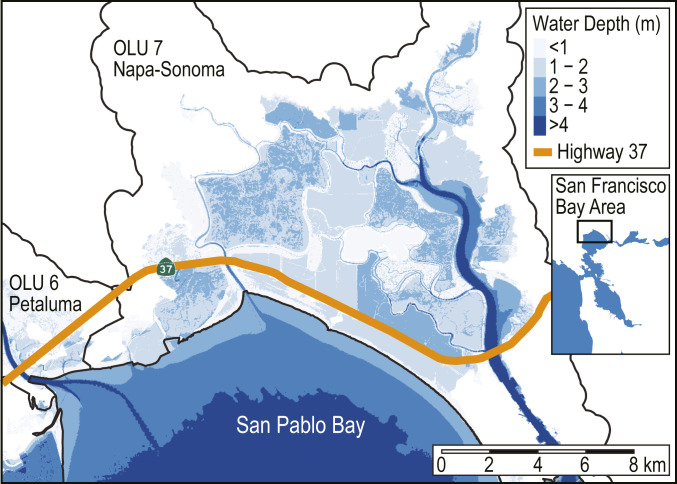
Highway 37, a transportation corridor of regional importance, spanning the OLU 7 (Napa–Sonoma) shoreline. Highway 37 is susceptible to SLR-induced flooding and will need to be adapted to prevent future disruptions, either by building the road on top of a raised levee or embankment or rebuilding it as a causeway. The choice of adaptation strategy will influence local and regional inundation and associated economic damages. Flooding caused by the 100-cm SLR scenario with existing shorelines is shown for reference.

From a project-level perspective, understanding flood externalities can help enhance cost–benefit analyses. A specific example from San Francisco Bay is the case of Highway 37 (Fig. 6), which runs along the northern shoreline of San Pablo Bay and connects two major thoroughfares in the region: Interstate 80 and Highway 101. More than half of the length of Highway 37 runs along the OLU 7 shoreline. Segments of this road already experience flooding during high-water events, and the state transportation agency, Caltrans, is considering adaptation alternatives to mitigate the effects of future flooding. The alternatives that are being considered include 1) building the road on top of a raised levee or embankment, estimated to cost $650 million, or 2) constructing a causeway that maintains tidal exchange between the bay and marshlands, estimated to cost $2.2 to 2.5 billion (40). These adaptation options can be seen as proxies for the two possible shoreline strategies examined in this study, including protecting the shoreline (Alternative 1: levee scenario) or maintaining flood pathways between the bay and the surrounding landscape (Alternative 2: causeway scenario). Although Alternative 2 would cost nearly four times as much to build as Alternative 1, the economic analysis presented here suggests that building a barrier along the OLU 7 shoreline could lead to a net increase of $293 million in damages across the bay at 200 cm of SLR due to the loss of flood storage space and induced flooding elsewhere. This estimate only captures damage to buildings at the highest annual tidal flood level and is not a probabilistic estimate of repetitive damage, which would likely lead to higher damages for any given SLR scenario. In addition, it does not include damage to other infrastructure systems (e.g., transportation, water, and energy) or land use types (e.g., agriculture) that will also be affected by flooding (31, 41). Even with these caveats, our results demonstrate that these damage externalities may be a substantial contributor to the overall cost–benefit analysis of proposed infrastructure alternatives and should not be neglected when evaluating and selecting infrastructure adaptation strategies.

Estimates of baywide change in damages due to shoreline protection provide insight into potential opportunities for strategic regional adaptation planning. In most cases, protecting an OLU leads to a net reduction in aggregate damages across the region (Fig. 4*F*), although individual OLUs may experience increased losses. For example, while protecting the OLU 25 shoreline leads to higher damages in other South Bay OLUs, the net regional damage reduction from shoreline protection still exceeds $1 billion in all SLR scenarios (*SI Appendix*, Table S2), highlighting the economic importance of this area. In cases such as this, compensation for communities that experience negative externalities is a possible solution (42), considering the high net benefit of shoreline protection. In some cases, however, shoreline protection leads to a net increase in damages across the entire region. For example, protecting OLU 7 causes up to $293 million in regional net damages at 200 cm of SLR, impacting both San Pablo Bay and South Bay, where total replacement values are generally the highest. Shoreline protection in OLU 21 also leads to a regional net increase in damages up to $194 million at 200 cm of SLR (*SI Appendix*, Table S2). Protecting OLUs 7 and 21, which are both classified as wide alluvial valleys, is thus difficult to justify from a regional economic perspective; instead, strategic flooding in these areas could provide substantial regional benefits by avoiding the negative economic externalities associated with shoreline protection. A transfer of development rights program that allows property owners to sign over their development rights for a portion of the proceeds from development elsewhere could be a mechanism that allows already densely developed areas to incentivize communities in wide alluvial valleys to avoid further development and allow strategic flooding to reduce flood levels throughout the bay. Importantly, the damage estimates we report here do not include the cost of construction and maintenance of armoring, nor do they include the potential degradation of coastal habitats (25) and loss of recreation, fisheries, and other ecosystem services that may influence the net benefits and costs of armoring (24, 43).

**Fig. 7.**
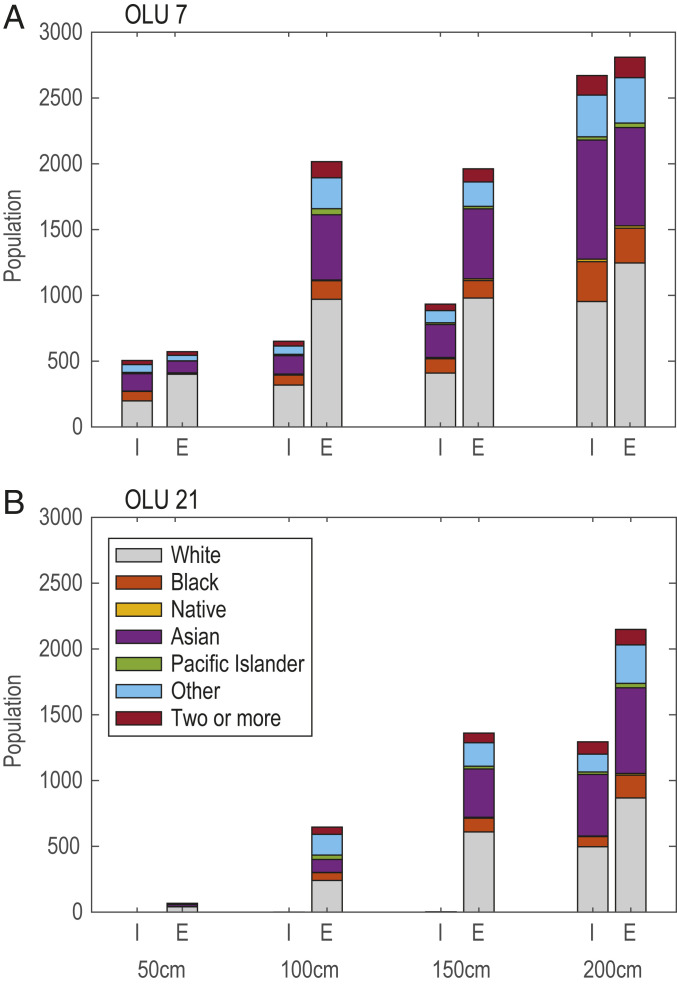
Total number and racial composition of people affected by the decision to strategically flood (*A*) OLU 7 and (*B*) OLU 21. For each SLR scenario, the left column (I = internal) represents the people living in OLU 7 or 21 who experience flooding as a result of this decision, while the right column (E = external) represents people living in other OLUs who avoid flooding.

There are, of course, other related factors that may influence the decision about protecting specific shoreline segments, including protection of vulnerable populations, agricultural areas, places of historical or cultural significance, and critical infrastructure assets of regional importance. For example, Fig. 7 shows the magnitude and demographic breakdown of the population affected by flooding when (*A*) OLU 7 and (*B*) OLU 21 are allowed to strategically flood, as suggested above. For each SLR scenario, the left column represents the people living in OLU 7 or 21 who experience flooding as a result of this decision, while the right column represents people living in other OLUs who avoid flooding. Strategic flooding of OLU 21 leads to protection of people throughout the bay at 50 cm, 100 cm, and 150 cm of SLR without flooding local residents. At 200 cm of SLR, strategic flooding leads to an increase in the flooded population within OLU 21. However, both with respect to the total number of people flooded and their racial composition, allowing flooding in OLU 21 provides benefits for more people of all races across the bay. Thus, the decision to allow OLU 21 to strategically flood to mitigate external impacts could be justified by both the damage and population data. However, individuals, communities, and decision-makers within OLU 21 would likely object to sacrificing local assets for the benefit of the broader community within the bay, even if compensated. Inclusive discussions among multiple stakeholders and decision-makers would certainly be a critical step in evaluating and implementing any such strategy.

In contrast, allowing OLU 7 to strategically flood at 50 cm of SLR causes flooding for 500 people (61% Black, indigenous, or people of color [BIPOC]) in OLU 7 while avoiding flooding for 570 people (30% BIPOC) elsewhere. At 100 cm and 150 cm of SLR, the number of people outside OLU 7 who benefit from strategic flooding in OLU 7 outweighs the number of people in OLU 7 who are affected, with comparable racial composition between both groups. However, at 200 cm of SLR, the flooded population in OLU 7 (2,670 people, 64% BIPOC) is once again similar in magnitude to the population that avoids flooding elsewhere (2,810 people, 56% BIPOC) and includes a higher percentage of BIPOC residents, who often have fewer resources to prepare for, respond to, and recover from natural hazards such as flooding (44). In this case, accounting for the number of residents internal to OLU 7 that experience flooding and the potential disparate impacts on the BIPOC population may lead to alternative decisions about shoreline adaptation in OLU 7. As this example illustrates, augmenting information about physical and economic externalities with estimates of associated human impacts can provide an additional means through which to evaluate proposed shoreline adaptation projects and to inform more equitable risk reduction.

The work summarized here is an important first step toward understanding previously uncounted regional damage interactions and thus fills a critical information gap in the understanding of shoreline protection and its consequences within San Francisco Bay. However, internalizing this information into decision making will require overcoming the “governance gap” that separates the scale of decision-making from the scale of the threat of SLR (7). Currently, the San Francisco Bay Area lacks a mechanism to reorient smaller-scale planning toward a coordinated, regional focus across jurisdictions. Possible avenues to address the gap include expanding the authority of existing regional planning and permitting agencies, such as the San Francisco Bay Conservation and Development Commission, or developing a collaborative management structure composed of multiple agencies working together to implement a regional vision, as proposed by the recent Bay Adapt initiative (45). Given the sizable potential flood damage externalities observed in this study, coordinated action is more likely to succeed if incentives are aligned to address disparate impacts across parties. Direct transfer payments as mentioned above may be an option to compensate areas that are strategically allowed to flood to reduce damages elsewhere; this is analogous to other payment for environmental service programs like water funds that preserve upstream land to ensure downstream water quantity and quality (46). A more targeted approach could be modeled after the Measure AA parcel tax, which funds restoration of the San Francisco Bay shoreline by taxing all parcels in the nine counties that border the bay $12 annually for 20 y. By raising funding for SLR adaptation at the regional level and tying it to development density, projects and policies could be prioritized based on regionally defined criteria and funded principally by developed areas that stand to benefit most.

Our results provide an initial estimate of the magnitude and distribution of flood damage externalities across communities when implementing coastal protection and strategic flood storage measures and can serve as a basis for transparent regional engagement that acknowledges these external costs. Although the OLU-scale shoreline protection scenarios presented here are not necessarily representative of likely SLR adaptation plans for the region, the results highlight how geomorphic factors, development density, and geographic location in the bay are likely to influence the regional impacts of shoreline protection projects. This information can support the evaluation and selection of actual adaptation plans and individual projects for the San Francisco Bay Area, which may include multiple simultaneous shoreline modifications that are implemented at smaller scales than examined here (e.g., sub-OLU). Similar analyses that consider other drivers of extreme water levels and associated patterns of flooding in addition to the tidal flooding mechanisms considered here would also help to inform adaptation decisions. Our approach can be extended to other coastal estuaries with low-lying, dense development, such as the Chesapeake Bay on the US East Coast or the Bohai Sea in China, which exhibit similar hydrodynamic feedbacks (27, 47, 48) and would presumably benefit from an analysis of interrelated economic outcomes from protection strategies. Accounting for the connectivity of local actions in coastal estuaries is a critical step toward identifying shoreline adaptation strategies that provide regional benefits while also mitigating unintended negative impacts.

## Materials and Methods

### Hydrodynamic Modeling.

We applied a two-dimensional depth-averaged hydrodynamic model of San Francisco Bay developed as part of the US Geological Survey’s (USGS) Coastal Storm Modeling System (CoSMoS) (37, 38). The model uses the Delft3D Flexible Mesh software (49), which applies a finite volume approach on an unstructured grid to solve the governing shallow water equations

$$\frac{\delta h}{\delta t}+\frac{\delta uh}{\delta x}+\frac{\delta vh}{\delta y}=0$$

$$\frac{\delta u}{\delta t}+u\frac{\delta u}{\delta x}+v\frac{\delta u}{\delta y}=-g\frac{\delta h}{\delta x}+\nu \left(\frac{\delta^{2}u}{\delta x^{2}}+\frac{\delta^{2}u}{\delta y^{2}}\right)-\frac{1}{C^{2}}\frac{g}{h}\|u\|u$$

$$\frac{\delta v}{\delta t}+u\frac{\delta v}{\delta x}+v\frac{\delta v}{\delta y}=-g\frac{\delta h}{\delta y}+\nu \left(\frac{\delta^{2}v}{\delta x^{2}}+\frac{\delta^{2}v}{\delta y^{2}}\right)-\frac{1}{C^{2}}\frac{g}{h}\|v\|v,$$

where h is the water depth, u and v are the depth-averaged velocities, g is the gravitational acceleration, ν is the viscosity, C is the drag coefficient, and x, y, and t are the space and time coordinates. Wetting and drying is accomplished by adding or removing grid points from the flow domain based on a threshold flood depth. Spatially variable roughness is applied using the Manning roughness formulation.

The model domain included San Francisco Bay and upstream channels in the Sacramento–San Joaquin Delta and extended offshore to the −1,500-m depth contour (*SI Appendix*, Fig. S3). Grid cells ranged in size from approximately 3 km in offshore areas to less than 50 m in overland areas. We used seamless topography and bathymetry data available at 2-m horizontal resolution from the USGS Coastal National Elevation Database (50) across the model domain. We further delineated existing shoreline protection features, such as engineered levees, floodwalls, berms, and embankments, in areas where the grid resolution was not fine enough to capture these features. Elevation data for these structures was extracted from the San Francisco Estuary Institute’s San Francisco Bay Shore Inventory database (32).

We forced the model at the oceanic boundary with January 2010 water levels and currents extracted from Oregon State University’s TPXO8 tidal model for eight harmonic constituents (M_2_, S_2_, N_2_, K_2_, K_1_, O_1_, P_1_, and Q_1_) (51). For each SLR scenario we added an additional tidal component with zero frequency and amplitude equal to the SLR increment (i.e., 50, 100, 150, and 200 cm). We applied historical discharge data for the Sacramento and San Joaquin Rivers as point inflows into the model. We did not include meteorological forcing in the simulations because the focus of the study is on tidally driven interactions with the shorelines. Outputs from the simulations represent the inundation that would occur at high tide during a spring tide cycle, which persists for approximately 2 wk each month. This results in permanent flooding in some low-lying areas and shorter-duration (minutes to hours) but frequent (multiple days per month) flood disruptions at higher elevations.

### Shoreline Scenarios.

We developed the shoreline scenarios from the OLU boundary delineation conducted by ref. 35 for the San Francisco Bay Area. Briefly, ref. 35 divided the bayshore broadly by geomorphic type, including wide alluvial valleys, alluvial fans and alluvial plains, and headlands and small valleys. They then further delineated the lateral boundaries between individual OLUs using major watershed boundaries or the apex points of major headlands and alluvial fans. In the cross-shore direction, OLUs extend from the offshore point where wind-driven waves are capable of mobilizing sediment to the inland extent of the 500-cm SLR scenario plus a 500-m transitional zone.

We implemented protection scenarios for each OLU shoreline individually in the hydrodynamic model using infinitely high impermeable walls. The walls generally follow the coastal boundary of each OLU, as well as the lateral boundaries up to the 200-cm SLR flooding extent modeled using existing shorelines. This prevents flooding between the protected OLU and its neighbors along overland flow pathways.

For each scenario, we calculated the change in inundation volume across the land surface in each OLU using the integral

$$V_{OLU}=\int_{A_{OLU}}\Delta h\;dA,$$

where $V_{OLU}$ is the inundation volume, $A_{OLU}$ is the surface area of the OLU, and Δh is the change in water depth in each grid cell as compared to the existing shoreline scenario for that amount of SLR.

### Economic Damages.

We simulated flood damages using the expected damage function methodology (52), estimating both the expected repair cost to flooded properties and the replacement cost of damaged building contents under the baseline no-intervention condition and all protection and SLR scenarios. Using this approach, the change in repair cost between a baseline scenario and protection scenario provides an estimate of compensating variation, or the social welfare gain/loss, associated with that protection scenario (53). This assumes risk neutrality of property owners and would underestimate the change in social welfare if affected owners were risk averse. We conducted this analysis for structures across the San Francisco Bay region represented in the Federal Emergency Management Agency’s (FEMA’s) HAZUS 2015 General Building Stock database, a nationwide spatially explicit inventory of structures classified by occupancy type. The spatial resolution of this dataset is the census block, and as such the expected damage function here is a lumped model where all structure classes are assumed to be evenly spread across the census block. We reconstructed aggregate structure and content repair/replacement costs by occupancy class for each census block outside of the HAZUS software following guidance in FEMA’s HAZUS 3.2 release notes (39). We derived total repair cost values at risk to SLR over the next 150 y by aggregating over all census blocks across OLUs, consistent with the risk profile definition used to create the OLUs (35). We calculated the total population at risk by aggregating across OLUs based on the Environmental Protection Agency’s 30-m dasymetric population map for the coterminous United States available from EnviroAtlas (54).

We assessed flood damages for each occupancy class across census blocks via depth-damage functions that relate flood depth to repair costs, as a fraction of total building replacement cost. From HAZUS, we extracted appropriate functions for all structure classes, as developed by FEMA and the US Army Corps of Engineers using empirical data from past flood events (55, 56). Census blocks are generally less than 3 ha, but in less densely populated areas they can be much larger and in all cases in the study area were larger than the resolution of the flood raster. To deal with variation in flood depth and nonlinear depth-damage functions to produce a single estimate of repair cost for each census block we randomly sampled 100 cells from the flood map within each census block and estimated repair costs across all occupancy types for each draw. From this we derived summary statistics for aggregate repair costs across occupancy classes for each census block and reported on sample means.

Modeling was treated as a one-off tidal flood event under each SLR scenario and did not account for repeat flood events. All else being equal, this significantly underpredicts long-term value estimates. We examined only economic damages to buildings and did not incorporate other infrastructure systems (e.g., transportation, water, and energy) or land-use types (e.g., agriculture), which will also be affected by flooding and contribute to economic damages (31, 41). Crop agriculture is a small portion of land by area, even in wide alluvial valleys (*SI Appendix*, Fig. S2), so we do not expect large systematic damage underestimates. We did not account for changes in socioeconomic development or population distribution over time (57), which could bias results depending on their future trajectory. While our analysis focused on property replacement values, protection of vulnerable populations may be a priority for communities but may be undervalued through traditional property value–based analyses such as the one presented here (58, 59).

### Population at Risk in Bays and Estuaries.

We defined population at risk from SLR and flooding here as those living adjacent to the shoreline at less than 10 m elevation, excluding areas that would not be hydrologically connected to the coast, consistent with prior work estimating SLR risk in what has been termed the “Low Elevation Coastal Zone” (2, 60). Global population in 2020 was mapped using WorldPop (61), and global elevation data were sourced from the Consultative Group on International Agricultural Research’s hole-filled Shuttle Radar Topography Mission global digital elevation model (62). The digital elevation model was reprocessed to identify areas below 10 m in elevation that are contiguous with the coastline, using the public domain World Data Bank II global shoreline vector layer as the reference coastline. This layer was then used to extract the global population that met these criteria. Finally, using a globally mapped typology of nearshore coastal systems (63), we extracted populations nearest to coastal systems defined as predominantly tidally influenced (class 2) to estimate total population at risk in this nearshore system. This process estimates that 864 million people globally are at risk, and 468 million of these live closest to shorelines classified as tidally influenced bays and estuaries. The overall global exposure estimate of 867 million here is within 4% of the mean value of two prior studies that calculated this risk metric (2, 60).

To estimate population impacts for the shoreline protection scenarios modeled here, we extracted block-level population counts across the San Francisco Bay region from the 2010 decennial census (64). We calculated the proportion of each census block that was flooded under each shoreline scenario and then applied that value to the block-level population count to determine the number of people affected by flooding. This approach assumes that the population is evenly distributed throughout each census block, which could lead to biases in larger census blocks or in areas where residential development is concentrated in only part of a block. We then compared the population counts in each OLU for each protection scenario with the existing shoreline scenario at the same SLR to determine the number of people across the region who experience flooding or who obtain protective benefits as a result of the protective action.

## Supplementary Material

Supplementary File

## Data Availability

The data and code used in this analysis are available through the Dryad data repository at https://doi.org/10.5061/dryad.2z34tmpmb (65) and https://doi.org/10.5061/dryad.g79cnp5pt (66).
