# Supplementary material for: Economic evaluation of sea-level rise adaptation strongly influenced by hydrodynamic feedbacks
Source: Proc Natl Acad Sci U S A. 2021 Jul 12;118(29):e2025961118. doi: 10.1073/pnas.2025961118 (PMC8307291; doi:10.1073/pnas.2025961118)
Supplement: Supplementary File [file pnas.2025961118.sapp.pdf]

PNAS
www.pnas.org

Supplementary Information for

Economic evaluation of sea-level rise adaptation strongly influenced by hydrodynamic feedbacks

Michelle A. Hummel, Robert Griffin, Katie Arkema, Anne D. Guerry

Michelle A. Hummel
E-mail: michelle.hummel@uta.edu

This PDF file includes:

Figs. S1 to S3
Tables S1 to S3
SI References

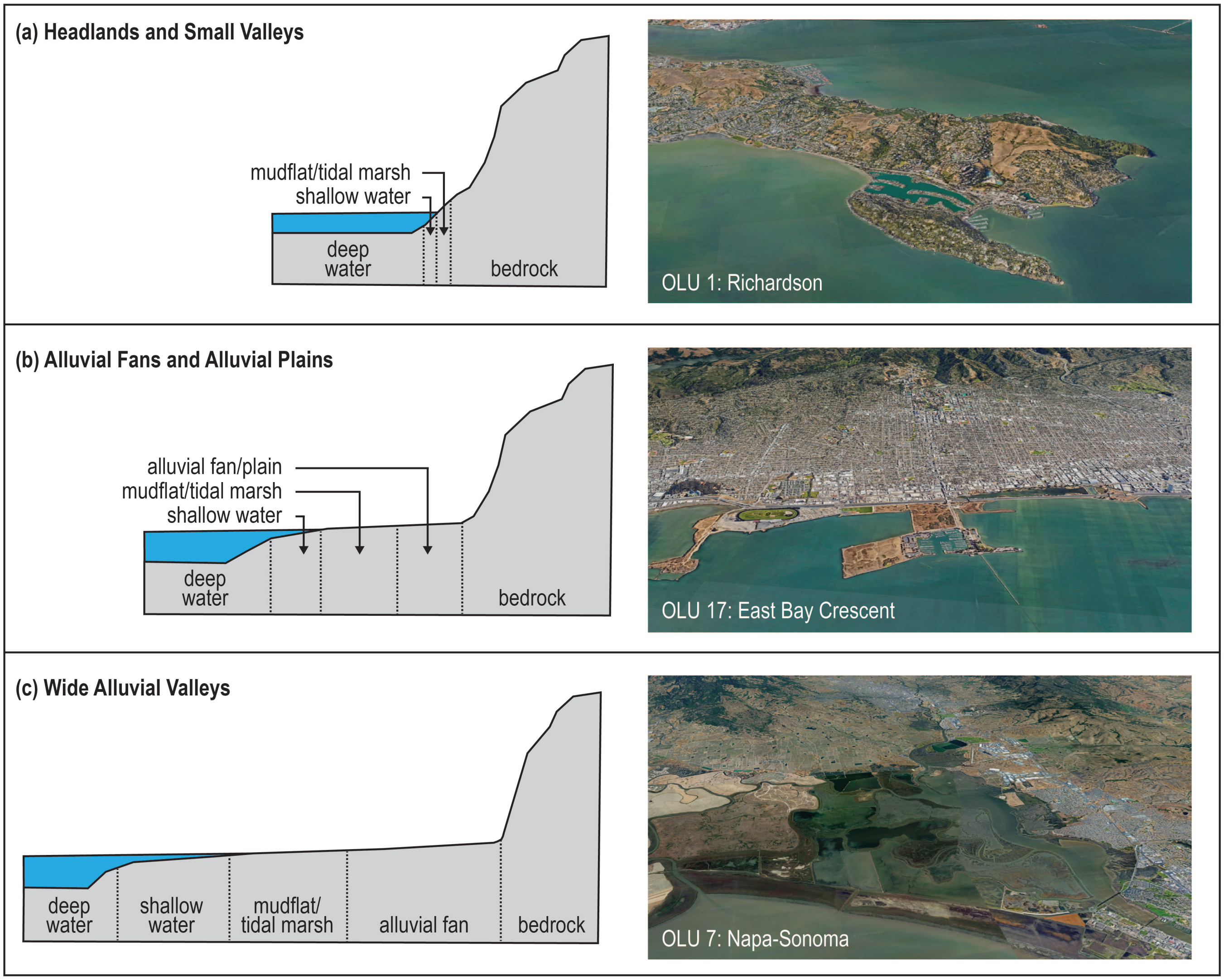

Fig. S1. OLU geomorphic types. (a) Headlands and Small Valleys have narrow baylands and steep slopes. (b) Alluvial Fans and Alluvial Plains have baylands of intermediate width and moderate slopes. (c) Wide Alluvial Valleys have wide baylands and gradual slopes. An example OLU from the San Francisco Bay Area is pictured for each geomorphic type. Modified from (1).

Table S1. Statistical significance values for Tukey Honest Significant Difference test for pairwise comparisons across geomorphic classes. Values are provided for external, internal, and net damages and flood volumes across all SLR scenarios.

Impact	SLR	Scenario Comparison	Damage P-Value	Volume P-Value
External	50cm	Headlands and Small Valleys-Alluvial Fans and Alluvial Plains	0.69	0.21
		Wide Alluvial Valleys-Alluvial Fans and Alluvial Plains	0.59	0.94
		Wide Alluvial Valleys-Headlands and Small Valleys	0.96	0.17
	100cm	Headlands and Small Valleys-Alluvial Fans and Alluvial Plains	0.92	0.21
		Wide Alluvial Valleys-Alluvial Fans and Alluvial Plains	0.58	0.03
		Wide Alluvial Valleys-Headlands and Small Valleys	0.78	0.00
	150cm	Headlands and Small Valleys-Alluvial Fans and Alluvial Plains	0.09	0.01
		Wide Alluvial Valleys-Alluvial Fans and Alluvial Plains	0.74	0.02
		Wide Alluvial Valleys-Headlands and Small Valleys	0.03	0.00
	200cm	Headlands and Small Valleys-Alluvial Fans and Alluvial Plains	0.22	0.01
		Wide Alluvial Valleys-Alluvial Fans and Alluvial Plains	0.53	0.07
		Wide Alluvial Valleys-Headlands and Small Valleys	0.04	0.00
Internal	50cm	Headlands and Small Valleys-Alluvial Fans and Alluvial Plains	0.19	0.88
		Wide Alluvial Valleys-Alluvial Fans and Alluvial Plains	0.32	0.00
		Wide Alluvial Valleys-Headlands and Small Valleys	1.00	0.00
	100cm	Headlands and Small Valleys-Alluvial Fans and Alluvial Plains	0.15	0.81
		Wide Alluvial Valleys-Alluvial Fans and Alluvial Plains	0.34	0.00
		Wide Alluvial Valleys-Headlands and Small Valleys	0.97	0.00
	150cm	Headlands and Small Valleys-Alluvial Fans and Alluvial Plains	0.16	0.75
		Wide Alluvial Valleys-Alluvial Fans and Alluvial Plains	0.35	0.00
		Wide Alluvial Valleys-Headlands and Small Valleys	0.97	0.00
	200cm	Headlands and Small Valleys-Alluvial Fans and Alluvial Plains	0.11	0.72
		Wide Alluvial Valleys-Alluvial Fans and Alluvial Plains	0.25	0.00
		Wide Alluvial Valleys-Headlands and Small Valleys	0.98	0.00
Net	50cm	Headlands and Small Valleys-Alluvial Fans and Alluvial Plains	0.26	0.98
		Wide Alluvial Valleys-Alluvial Fans and Alluvial Plains	0.32	0.00
		Wide Alluvial Valleys-Headlands and Small Valleys	1.00	0.00
	100cm	Headlands and Small Valleys-Alluvial Fans and Alluvial Plains	0.15	0.89
		Wide Alluvial Valleys-Alluvial Fans and Alluvial Plains	0.26	0.00
		Wide Alluvial Valleys-Headlands and Small Valleys	1.00	0.00
	150cm	Headlands and Small Valleys-Alluvial Fans and Alluvial Plains	0.23	0.83
		Wide Alluvial Valleys-Alluvial Fans and Alluvial Plains	0.29	0.00
		Wide Alluvial Valleys-Headlands and Small Valleys	1.00	0.00
	200cm	Headlands and Small Valleys-Alluvial Fans and Alluvial Plains	0.14	0.79
		Wide Alluvial Valleys-Alluvial Fans and Alluvial Plains	0.18	0.01
		Wide Alluvial Valleys-Headlands and Small Valleys	0.99	0.00

Table S2. Total building replacement value and net damage (internal + external) from OLU protection scenarios, across SLR scenarios. All values in millions of 2010 USD.

		Total	Net Damages						Replacement
ID	Name	Replacement Cost	50cm	100cm	150cm	200cm	Geomorphic Type	Area (km^2)	Value per km^2
1	Richardson	5089.9	14.6	-117.6	-395.1	-411.4	Headlands and Small Valleys	24.3	209.3
2	Corte Madera	4050.2	-33.6	-368.6	-617.6	-634.1	Headlands and Small Valleys	22.8	177.6
3	San Rafael	4011.7	-8.4	-13.0	-982.3	-1222.6	Headlands and Small Valleys	17.7	226.4
4	Gallinas	2326.5	74.3	-70.2	-239.7	-218.3	Headlands and Small Valleys	25.2	92.1
5	Novato	3195.8	-132.5	-537.4	-604.2	-640.2	Headlands and Small Valleys	45.3	70.5
6	Petaluma	2486.4	-0.6	-63.4	-57.1	102.6	Wide Alluvial Valleys	103.7	24.0
7	Napa - Sonoma	8673.9	127.0	85.4	170.6	292.9	Wide Alluvial Valleys	275.3	31.5
8	Carquinez North	2375.0	69.2	-67.5	-35.9	59.7	Headlands and Small Valleys	18.0	131.9
9	Suisun Slough	4550.8	66.1	-103.5	-144.0	-123.4	Wide Alluvial Valleys	158.3	28.8
10	Montezuma Slough	29.6	113.0	-107.0	-71.3	84.8	Wide Alluvial Valleys	204.2	0.1
11	Port Chicago	993.1	90.8	-76.0	-96.5	-6.6	Alluvial Fans and Alluvial Plains	21.2	46.8
12	Walnut	1809.4	60.7	-47.8	-20.6	130.3	Wide Alluvial Valleys	41.9	43.2
13	Carquinez South	846.9	75.6	-12.9	-57.2	17.0	Headlands and Small Valleys	10.8	78.4
14	Pinole	2243.0	64.0	-104.6	-68.2	155.9	Headlands and Small Valleys	14.8	151.1
15	Wildcat	1360.2	82.7	-76.1	-67.1	52.7	Alluvial Fans and Alluvial Plains	21.3	63.7
16	Point Richmond	269.3	63.1	-85.9	-156.4	38.7	Headlands and Small Valleys	4.6	58.8
17	East Bay Crescent	8951.9	-33.6	-107.4	-168.0	-129.2	Alluvial Fans and Alluvial Plains	31.2	287.2
18	San Leandro	22851.1	30.5	-580.6	-1334.2	-2795.8	Alluvial Fans and Alluvial Plains	76.8	297.4
19	San Lorenzo	5133.5	-3.4	-189.9	-459.2	-574.5	Alluvial Fans and Alluvial Plains	33.4	153.9
20	Alameda	9837.2	53.2	-114.1	-68.7	-963.5	Alluvial Fans and Alluvial Plains	87.5	112.4
21	Mowry	3745.8	122.7	0.5	9.4	194.3	Alluvial Fans and Alluvial Plains	66.8	56.1
22	Santa Clara Valley	15375.6	-127.8	-444.4	-552.7	-945.8	Wide Alluvial Valleys	126.1	121.9
23	Stevens	5318.0	38.9	-502.3	-1303.2	-1573.4	Wide Alluvial Valleys	46.6	114.2
24	San Francisquito	2221.1	53.4	-342.2	-225.3	-420.6	Alluvial Fans and Alluvial Plains	13.8	160.5
25	Belmont - Redwood	11970.1	-1159.1	-2815.8	-4680.4	-5664.9	Alluvial Fans and Alluvial Plains	76.5	156.4
26	San Mateo	8199.2	-2236.9	-2769.5	-3016.7	-3695.1	Alluvial Fans and Alluvial Plains	23.1	355.5
27	Colma - San Bruno	8550.2	-142.9	-437.6	-882.4	-1233.1	Alluvial Fans and Alluvial Plains	30.8	277.5
28	Yosemite - Visitacion	3348.3	40.1	-103.8	-56.1	-56.4	Headlands and Small Valleys	16.1	208.2
29	Mission - Islais	28808.6	-17.5	-199.6	-466.4	-1421.3	Headlands and Small Valleys	22.2	1296.9
30	Golden Gate	5290.0	35.3	-162.1	-180.5	-208.2	Headlands and Small Valleys	6.1	860.8

Table S3. Change in internal and external flooded population from OLU protection scenarios, across SLR scenarios.

		Change in Internal Flooded Population				Change in External Flooded Population			
ID	Name	50cm	100cm	150cm	200cm	50cm	100cm	150cm	200cm
1	Richardson	-314	-1,176	-2,067	-2,860	-19	-91	-104	-109
2	Corte Madera	-801	-2,877	-4,800	-6,486	-16	-386	60	-39
3	San Rafael	-79	-221	-13,004	-13,387	19	-6	13	-26
4	Gallinas	-6	-27	-2,985	-3,422	25	-108	138	67
5	Novato	-1,373	-2,243	-3,454	-4,401	-8	-129	316	296
6	Petaluma	-460	-528	-726	-88	247	413	494	474
7	Napa - Sonoma	-505	-651	-934	-2,671	572	2,016	1,962	2,810
8	Carquinez North	-47	-82	-117	-157	183	413	285	182
9	Suisun Slough	-5	-276	-2,731	-3,276	6	20	-46	-28
10	Montezuma Slough	-33	-52	-58	-62	3	303	5	-39
11	Port Chicago	-1	-89	-176	-1,564	87	45	77	-5
12	Walnut	0	-1	-2	-4	50	91	194	53
13	Carquinez South	-27	-38	-50	-64	36	72	121	16
14	Pinole	-13	-19	-30	-345	1	-3	15	-6
15	Wildcat	-4	-7	-23	-50	23	63	132	-31
16	Point Richmond	-8	-11	-25	-42	-22	-444	6	-45
17	East Bay Crescent	-33	-51	-305	-1,898	-11	-6	20	-28
18	San Leandro	-298	-6,315	-19,181	-34,472	80	596	864	157
19	San Lorenzo	-230	-2,415	-4,752	-10,657	32	247	-693	760
20	Alameda	-45	-265	-2,295	-25,717	36	537	980	2,148
21	Mowry	0	-1	-2	-1,294	61	646	1,360	2,149
22	Santa Clara Valley	-945	-2,412	-7,066	-12,975	266	1,733	2,210	5,898
23	Stevens	-1	-6,696	-10,181	-11,876	24	766	872	1,012
24	San Francisquito	-786	-4,485	-6,496	-8,003	37	-614	-74	-1,077
25	Belmont - Redwood	-28,041	-41,050	-49,251	-56,495	1,593	1,777	1,737	3,621
26	San Mateo	-17,645	-28,058	-39,040	-41,819	-27,085	-5,515	-800	334
27	Colma - San Bruno	-148	-307	-1,736	-3,782	60	-2	701	-17
28	Yosemite - Visitacion	-37	-43	-45	-53	300	-448	-19	-61
29	Mission - Islais	-2	-255	-2,303	-8,881	-42	-317	-145	-1,145
30	Golden Gate	0	-2	-271	-1,230	228	-515	-357	-1,459

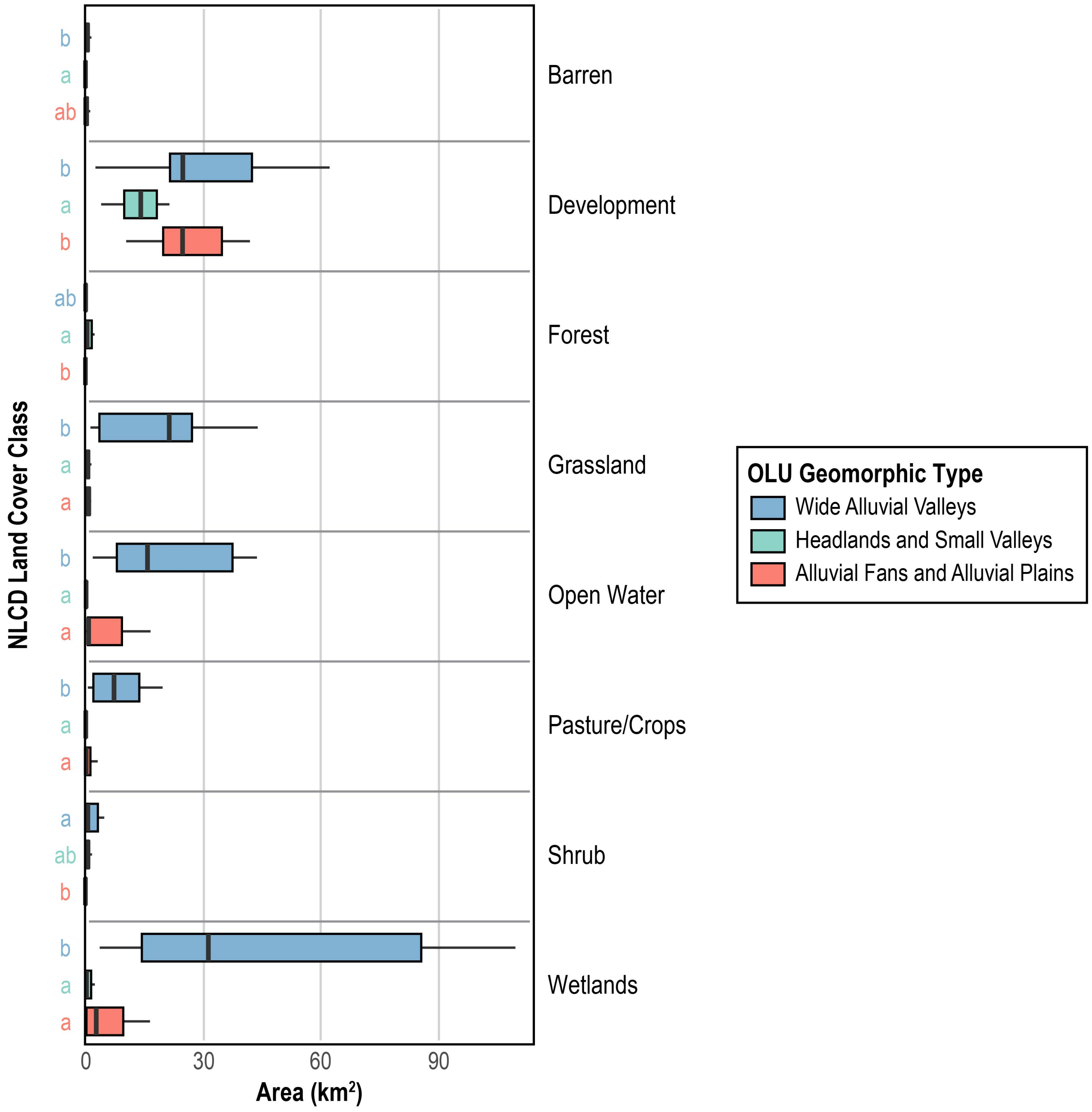

Fig. S2. Land cover classes within geomorphic types, based on the 2016 National Land Cover Database. Letters represent statistically similar mean values for all pairwise comparisons across geomorphic classes within a landcover type, based on a Tukey Honest Significant Difference test at 5% significance level.

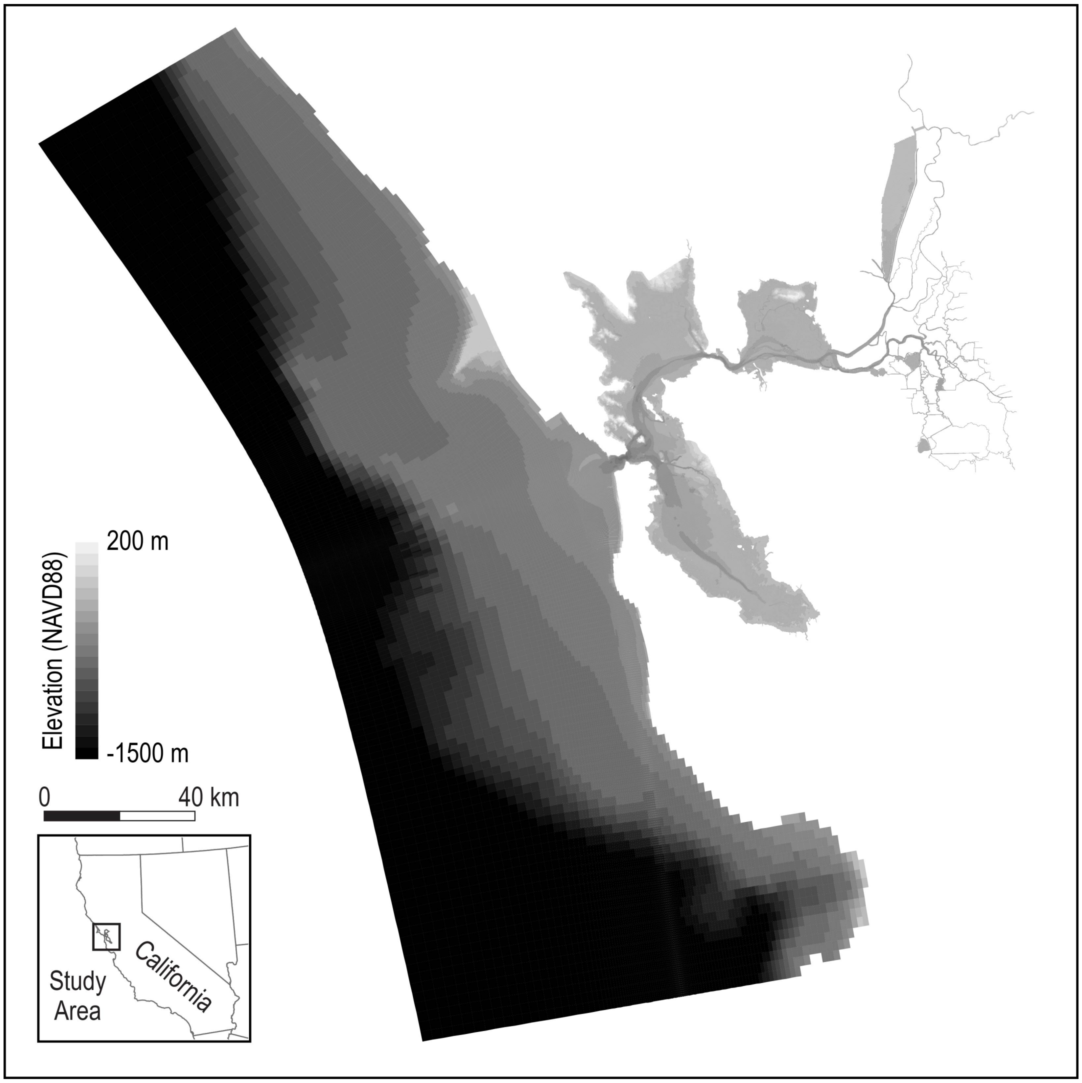

Fig. S3. Computational domain and topography/bathymetry for San Francisco Bay hydrodynamic model.

References

1. J Beagle, et al., San Francisco Bay Shoreline Adaptation Atlas, (San Francisco Estuary Institute and San Francisco Bay Area Planning and Urban Research Association), Technical report (2019).
